# The role of CSDE1 in translational reprogramming and human diseases

**DOI:** 10.1186/s12964-019-0496-2

**Published:** 2020-01-27

**Authors:** Ao-Xiang Guo, Jia-Jia Cui, Lei-Yun Wang, Ji-Ye Yin

**Affiliations:** 1 Department of Clinical Pharmacology, Xiangya Hospital, Central South University, Changsha, 410078 People’s Republic of China; 2 Institute of Clinical Pharmacology, Central South University; Hunan Key Laboratory of Pharmacogenetics, Changsha, 410078 People’s Republic of China; 3 Engineering Research Center of Applied Technology of Pharmacogenomics, Ministry of Education, 110 Xiangya Road, Changsha, 410078 People’s Republic of China; 4 National Clinical Research Center for Geriatric Disorders, 87 Xiangya Road, Changsha, 410008 Hunan People’s Republic of China; 5 Hunan Provincial Gynecological Cancer Diagnosis and Treatment Engineering Research Center, Changsha, 410078 People’s Republic of China; 6 Hunan Key Laboratory of Precise Diagnosis and Treatment of Gastrointestinal Tumor, Changsha, 410078 People’s Republic of China

**Keywords:** CSDE1, UNR, Reprogramming, Translation initiation, RNA binding protein, Cancer

## Abstract

**Abstract:**

CSDE1 (cold shock domain containing E1) plays a key role in translational reprogramming, which determines the fate of a number of RNAs during biological processes. Interestingly, the role of CSDE1 is bidirectional. It not only promotes and represses the translation of RNAs but also increases and decreases the abundance of RNAs. However, the mechanisms underlying this phenomenon are still unknown. In this review, we propose a “protein-RNA connector” model to explain this bidirectional role and depict its three versions: sequential connection, mutual connection and facilitating connection. As described in this molecular model, CSDE1 binds to RNAs and cooperates with other protein regulators. CSDE1 connects with different RNAs and their regulators for different purposes. The triple complex of CSDE1, a regulator and an RNA reprograms translation in different directions for each transcript. Meanwhile, a number of recent studies have found important roles for CSDE1 in human diseases. This model will help us to understand the role of CSDE1 in translational reprogramming and human diseases.

Video Abstract

**Graphical abstract:**

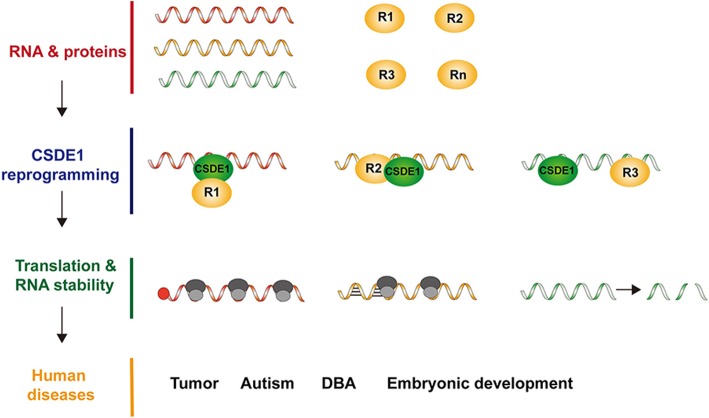

## Background

Gene expression is regulated at two major levels: transcription and translation [1]. Translation is the process of transforming intracellular mRNA into protein, and translational homeostasis is critical for cells to properly perform their functions [2]. When the cellular internal or external environment is changed, the translation of a subset of mRNAs is reprogrammed to determine cell fate [3–5]. As the final step in gene expression, translation is more directly and quickly regulated compared with other steps. Thus, translational reprogramming is critical for cells to rapidly adapt to environmental changes.

Evidence indicates that CSDE1, also known as upstream of N-RAS (UNR), plays an important role in translational reprogramming. It is an RNA-binding protein (RBP) that contains five cold shock domains and is mainly expressed in the cytoplasm [6–9]. CSDE1 plays an important role in a wide range of biological processes, including the cell cycle [10], apoptosis [11], differentiation [12] and dosage compensation [13] (Table 1). CSDE1 controls translation initiation of some mRNAs and affects their expression. Furthermore, CSDE1 determines the fate of mRNAs by changing their stability and abundance [26]. Thus, it is a critical factor during the translational reprogramming process. Furthermore, CSDE1 plays a dual role in regulating translation and the abundance of RNAs. However, the mechanisms underlying this phenomenon are still unknown.

**Table 1:** Reprogramming role of CSDE1 in biological processes and diseases

Biological processes and diseases	The role of CSDE1	References
Biological processes		
Cell cycle	Promotion	[10]
Apoptosis	Promotion	[11]
Differentiation	Repression	[12]
Dosage compensation (male)	Promote DCC assembly	[14]
Dosage compensation (female)	Repress DCC formation	[13]
Tumors		
Melanoma	Oncogene	[15]
Colorectal cancer	Oncogene	(98)
Glioma	Oncogene	[16]
Breast cancer	Oncogene	[17]
PCCs &PGLs	Tumor suppressor	[18]
Oral squamous cell carcinoma	Tumor suppressor	[19]
Pancreatic ductal adenocarcinoma	Prognostic biomarker	[20]
Epithelial ovarian cancer	Platinum resistance gene	[21]
Other diseases		
Autism spectrum disorder	Loss of function mutation	[22]
Diamond-Blackfan anemia	Low expression	[23]
Embryonic development	Prevent ESC differentiation	[24, 25]

In this review, we focus on the bidirectional reprogramming functions of CSDE1 and propose a theoretical model to explain the underlying mechanism. This mechanism may contribute to understanding the role of CSDE1 in human diseases. CSDE1 structure, expression, activity and regulation have been discussed in previous publications [27, 28]. They will not be discussed in this review.

## Bidirectional translational reprogramming

### Promoting and repressing cap-independent translation initiation

For translation control, the initiation stage is the rate-limiting step [29]. In eukaryotes, most translation initiation events rely on binding of the cap-binding complex at the 5’ end of mRNA [30, 31]. However, under the conditions of impaired canonical cap-dependent translation, cap-independent translation is required for cell survival and stress recovery [32–34]. During this process, 40S ribosomes can be directly recruited via an internal ribosome entry site (IRES) element to the 5’ untranslated region (UTR) of mRNA [35, 36]. IRES trans-acting factors (ITAFs) are necessary for helping to recruit 40S ribosomes to promote translation. CSDE1 is a special ITAF [37]. It is involved in translation regulation mainly via promoting and repressing IRES-mediated cap-independent translation initiation. IRES elements exist in both eukaryotes and viruses [38]. CSDE1 can promote cap-independent mRNA translation initiation via IRES elements in both eukaryotes [39, 40] and viruses [41, 42].

In eukaryotes, IRES-mediated translation is widely found to be reprogrammed by CSDE1. Apoptosis is the process of programmed cell death. Apoptotic peptidase activating factor 1 (Apaf-1) is a key protein with the capacity to activate caspase 9 during the apoptotic process, and the translation initiation of Apaf-1 is primarily mediated by its IRES element [43]. The role of CSDE1 in promoting Apaf-1 IRES-mediated translation initiation has been thoroughly studied. In the rabbit reticulocyte lysate (RRL) system, Apaf-1 IRES activity was stimulated by adding purified CSDE1 protein [11]. In cells, Apaf-1 IRES activity was also significantly correlated with the expression level of CSDE1. Transfection of CSDE1 into cell lines (COS7, MRC5, SY5Y and BALB/c) increased Apaf-1 IRES activity by approximately 2.5-fold. However, transfection of CSDE1 into HeLa and HEK293 cells had no additional stimulation of IRES-independent translation [11], which could suggest that the translation stimulation of CSDE1 on Apaf-1 IRES is cell type specific. In addition, CSDE1 could bind to the Apaf-1 IRES to change its structure and permit the binding of other ITAFs. Through this mechanism, CSDE1 could further stimulate Apaf-1 IRES-dependent translation with the help of other protein regulators [11].

The cell cycle is the most important process for cell growth and proliferation. p58^PITSLRE^ is a PITSLRE protein kinase isoform that is essential for the cell cycle [44]. The translation of P58^PITSLRE^ was mediated by an IRES element in the PITSLRE mRNA. In HEK293T cells, CSDE1 barely bound to the PITSLRE IRES element, and there was nearly no expression of p58^PITSLRE^ in the G1 phase [40]. However, during the G2/M phase, when global translation (especially cap-dependent translation) was repressed, p58^PITSLRE^ expression was greatly enhanced due to the significant increased binding of CSDE1 on its IRES element [10, 40]. Deletion of CSDE1 binding sites from the human PITSLRE cDNA decreased IRES activity by nearly 50% [40]. In addition, CSDE1 showed a positive effect on PITSLRE IRES activity in an RRL system [40]. Therefore, CSDE1 is necessary for PITSLRE IRES-dependent translational reprogramming during the cell cycle.

During tumourigenesis, c-myc mRNA is an oncogene involved in cell growth and death. It was reported that c-myc overexpression in various cells was mainly due to the aberrant activation of its IRES in the 5’UTR [45, 46]. CSDE1 could bind to the c-myc IRES element and have little or no effect on its activity in RRL [11]. However, CSDE1 greatly increased c-myc IRES activity in both RRL and HeLa cells by working together with other ITAFs, including poly (rC) binding protein 1 (PCBP1), poly (rC) binding protein 2 (PCBP2), heterogeneous nuclear ribonucleoprotein K (hnRNPK) and UNR-interacting protein (Unrip) [47]. Thus, CSDE1 is a positive regulator of c-myc IRES activity.

For viruses, CSDE1 also stimulates cap-independent translation initiation. Human rhinovirus (HRV) and poliovirus (PV) mainly utilize IRES-driven translation to promote their protein synthesis [41]. CSDE1 plays an important role in the translational control of these two viruses. Knocking out CSDE1 severely reduced HRV and PV IRES activity to one-tenth of the control group; this reduction occurs through specifically decreasing IRES activity without changing cap-dependent translation activity. In addition, adding back expression of CSDE1 in CSDE1^−/−^ mouse embryonic stem (ES) cells rescued the HRV and PV IRES activity to approximately 69 and 66%, respectively [41]. Thus, a number of studies have shown that CSDE1 could promote IRES-dependent translation initiation in both eukaryotes and viruses.

In contrast, CSDE1 can also repress cap-independent translation initiation of mRNA. One mRNA target is CSDE1 itself. The CSDE1 protein autoregulates its own expression by repressing IRES activity. In mouse ES cells, the activity of exogenous CSDE1 IRES was 2-fold higher in CSDE1^−/−^ cells than in CSDE1^+/−^ and CSDE1^+/+^ cells. In addition, transfection of exogenous CSDE1 reduced CSDE1 IRES activity by 60% [48]. Thus, CSDE1 can both promote and repress cap-independent translation initiation. It should be noted that these opposing functions can exist within the same cellular process. For example, during the cell cycle, CSDE1 can increase PITSLRE IRES activity and simultaneously decrease its own IRES activity. This indicates that the homeostasis of CSDE1 bidirectional reprogramming is important for its function.

### Promoting and repressing cap-dependent translation initiation

Although CSDE1 mainly reprograms cap-independent translation initiation, CSDE1 can also stimulate and repress cap-dependent translation initiation. In cap-dependent translation processes, eukaryotic translation initiation factor 4G (eIF4G) and poly(A)-binding protein 1 (PABP1) interact with each other to promote the circularization of terminal ribosomes from the 3’ end to the 5’ end [49–51]. Through enhancing the eIF4G-PABP1 interaction, CSDE1 increased the interaction between the 5’ cap and poly(A) tail to promote cap-dependent translation. Depletion of CSDE1 led to less eIF4G being pulled down by PABP1 from both HeLa and U2OS cells [52]. Additionally, a dual luciferase reporter assay showed that the cap-dependent translation initiation activity was reduced by 33% in CSDE1-depleted cells [52]. A recent study proposed another mechanism to explain how CSDE1 promoted cap-dependent translation initiation. It showed that CSDE1 defined a novel class of nucleoplasmic reticulum (NR) named CSDE1-NR, where CSDE1 foci were concentrated. CSDE1-NRs concentrated poly(A) RNAs, ribosomes and translation factors (such as eIF4E) to facilitate cap-dependent translation initiation. Based on ribopuromycylation method analysis, more than 80% of CSDE1-NRs were puromycin positive, which could be directly visualized through localized translation in the cancer cell line BeWo [24]. Through regulating translation factors, CSDE1 plays an active role in the cap-dependent translational reprogramming process.

On the other hand, the cap-dependent translation initiation of some specific mRNAs is frequently repressed by sequence-special RNA-binding proteins, which bind to response elements in the 5’UTR or 3’UTRs of RNA [29]. CSDE1 can repress the translation of PABP and male-specific lethal-2 (msl-2) by this mechanism. PABP protein autoregulated its expression by binding the adenine-rich autoregulatory sequence (ARS) in the 5’UTR of PABP mRNA, thus creating a negative feedback mechanism [53]. The binding of CSDE1 and insulin-like growth factor II mRNA binding protein-1 (IMP1) to the ARS element maximally repressed translation of PABP mRNA in a cell-free RRL system [54]. CSDE1 and IMP1 stalled the movement of the 40S pre-initiation complex on the PABP 5’UTR [51, 54]. Msl-2 was found to play a key role in dosage compensation of drosophila, which occurs by increasing transcription of the single male X chromosome by 2-fold in males and repressing it in females. This process requires the binding of the dosage compensation complex (DCC) to hundreds of sites along the male X chromosome, and msl-2 is the key component of the DCC [55, 56]. In females, CSDE1 was recruited by sex lethal (SXL) to msl-2 mRNA to repress its cap-dependent translation initiation [13]. Depleting CSDE1 restored the repressed translation, while the addition of recombinant CSDE1 could re-repress translation in a dose-dependent manner [14].

### Increasing and decreasing RNA abundance

RNA abundance directly influences protein production, so RNA abundance is a crucial component of translation control. The stability of RNA is one of the key factors determining RNA abundance. CSDE1 plays a bidirectional translational reprogramming role for RNAs by both increasing and decreasing mRNA stability.

Existing studies indicate that CSDE1 could increase the abundance of some RNAs by maintaining their stability. CSDE1 tends to increase the RNA stability of some targeted RNAs including PTH, c-fos and lncBC200 via binding to the special element of theses RNAs. During the post-transcriptional regulation of parathyroid hormone (PTH) expression, some factors formed an RNA-binding complex on a defined cis-acting instability element in the 3’UTR to protect PTH mRNA from degradation [57]. As one key functional component of the stabilizing complex, CSDE1 contributes to the regulation of PTH mRNA abundance through attaching to the cis-acting instability element in the 3’UTR [58]. In HEK293 cells, overexpression of CSDE1 increased the full-length PTH mRNA abundance by more than 2-fold, while depletion of CSDE1 resulted in a decrease of PTH abundance [58]. Another mechanism comes from c-fos, which is a marker of neuronal activation [59]. Stability of c-fos mRNA is controlled by a special element major protein-coding-region determinant (mCRD). The mCRD-mediated mRNA turnover, can result in rapidly degrading aberrant mRNA [60]. CSDE1 plays a critical role in c-fos stability by binding to the mCRD. In NIH3T3 cells, knocking down CSDE1 decreased the rate of c-fos mRNA decay mediated by mCRD [61]. Overexpression of CSDE1 in NIH3T3 cells had a stabilizing effect on c-fos mRNA [61, 62]. In addition to mRNA, CSDE1 can increase long non-coding RNA (lncRNA) stability by binding to special elements as well. LncBC200 is a 200-nucleotide ncRNA that is normally highly expressed in the brain, but it is aberrantly expressed in various cancers [63]. In MCF-7 cells, CSDE1 could maintain the stability of lncBC200 by binding to the 3’ A-rich region, while knocking down CSDE1 reduced the half-life of lncBC200 by 40% and decreased its expression [64].

Regarding decreasing mRNA abundance, CSDE1 destabilizes a lot of mRNAs related with differentiation. GATA binding protein 6 (GATA6) is one of the earliest markers of the primitive endoderm (PrE) in the early mouse embryo, and it has a critical role in regulating PrE development [25]. Mouse GATA6 mRNA contains a long 3’UTR with two conserved purine-rich motifs at nucleotides 166–176 and 828–838 (downstream from the stop codon), and they are potential CSDE1-binding motifs. Deletion of CSDE1 in mouse ES cells increased GATA6 mRNA stability by approximately 25% [12]. By destabilizing GATA6 mRNA, CSDE1 repressed the expression of GATA6 and thus limited embryo stem cell differentiation in a dose-dependent manner. CSDE1 knockout ESCs spontaneously differentiated into PrE, and restoration of CSDE1 expression partially rescued the differentiation ability [12]. Apart from GATA6, CSDE1 also repressed the expression of many other mRNAs during the process of neural differentiation. Knocking down CSDE1 in human embryonic stem cells (hESCs) resulted in a significant increase of the level of many mRNA targets including FABP7, VIM and so on [65]. In addition, a lot of other CSDE1-bound targets were regulated by CSDE1 in the level of mRNA [15, 26].

## “Protein-RNA connector” mode

**Fig. 1:**
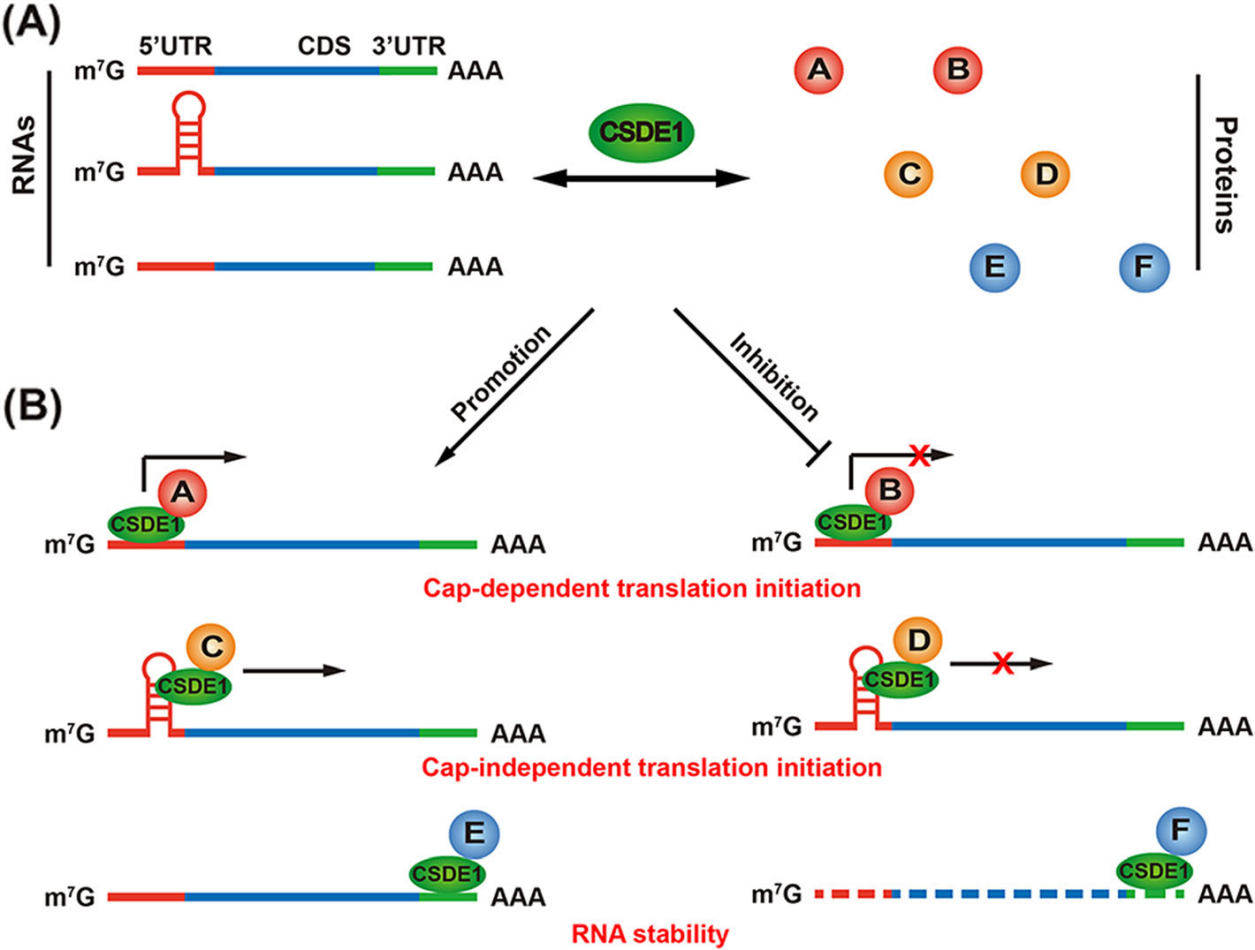
The bidirectional role of CSDE1 in translational reprogramming. (A) CSDE1 connects different protein regulators (A-F) with target RNAs to change the fate of RNAs. (B) The bidirectional role of CSDE1 in translational reprogramming. CSDE1 not only promotes and represses cap-dependent and cap-independent translation initiation of RNAs but also increases and decreases RNA abundance

The role of CSDE1 in translation regulation has been widely and thoroughly investigated [27, 28]. As discussed above, even within the same biological process, CSDE1 reprograms the translation of different mRNAs in completely opposite directions. The detailed underlying mechanism behind this dual function remains unclear. Based on previous investigations, we found that CSDE1 reprograms translation by acting as a bridge between RNAs and proteins. In most situations, CSDE1 regulates mRNA translation together with other protein partners instead of working alone. Furthermore, CSDE1 does not have regular RNA or protein binding partners. It binds a wide range of RNAs and connects to different protein regulators for each of them. Thus, CSDE1 changes the fate of RNAs by acting as a connector between RNAs and paired protein regulators. This “protein-RNA connector” model can be used to explain its bidirectional role in translational reprogramming (Fig. 1). Based on the specific regulator and the CSDE1 connecting mechanism, this model can be further separated into three forms: sequential connections, mutual connections and facilitating connections.

### Sequential connections

In the sequential connection mechanism, translational regulators cannot bind directly with RNA. They interact with targeted RNAs via binding to CSDE1. The protein regulator, CSDE1 and the RNA form a sequential connection model of “regulator-CSDE1-RNA”. CSDE1 acts as a bridge to connect RNAs and proteins that cannot bind directly to each other.

The function of serine/threonine kinase receptor associated protein (Strap) on RNAs can be explained by this mechanism. Strap is also named Unrip due to its interaction with CSDE1 [66]. Unrip had no inherent RNA binding capacity, but CSDE1 bridges Unrip with target RNAs [67]. One example is lncBC200, a long noncoding RNA that directly and indirectly interacts with CSDE1 and Unrip, respectively. CSDE1 binds to the 3′ A-rich region of lncBC200. Unrip indirectly binds to lncBC200 via heterodimerization with CSDE1. Immunoprecipitation assays showed that knocking down Unrip had a negligible impact on lncBC200 binding to CSDE1. However, knocking down CSDE1 in MCF-7 cells reduced Unrip binding on lncBC200 by more than 80% [64]. These results suggest the existence of a sequential connection of “Unrip-CSDE1-lncBC200”. Another example is HRV-2. Unrip and CSDE1 form a sequential connection of “Unrip-CSDE1-HRV2” to stimulate HRV-2 IRES activity and promote its translation initiation in MLE cells. Additional studies reported that Unrip regulated the translation of more CSDE1-bound transcripts, including hydroxymethylbilane synthase (Hmbs), eukaryotic translation initiation factor 4G3 (eIF4G3), poly(A) binding protein cytoplasmic 4 (PABPC4) and so on [68].

The sequential connection mode is applicable for translational regulators binding mRNA indirectly through CSDE1. In this mode, CSDE1 provides proteins with a platform for translational reprogramming. However, the mechanism is less reported.

### Mutual connections

For the translation regulators with the ability to bind RNA, CSDE1 can enhance the interaction between that protein and its target RNA via binding to both of them. The three then form a trimeric complex with internal mutual interaction to reprogram the translation or stability of bound RNAs. This mechanism has been widely studied.

One of the strongest pieces of evidence for this mechanism is that the CSDE1/SXL complex corepresses the translation of msl-2 in female drosophila. Two repressors, CSDE1 and SXL, can interact with each other, and both can bind to msl-2 mRNA. When repressing msl-2 translation, the female-specific RNA binding protein SXL binds to the 3’UTR of msl-2 mRNA and recruits CSDE1 to adjacent regulatory sequences [14, 69–71]. Then, CSDE1 formed a co-repressor complex with SXL to inhibit ribosome recruitment, which represses msl-2 mRNA translation [14, 70, 72]. The purine-rich sequence downstream of AGCACGUG (nucleotides 9–16) was sandwiched by the CSD1 domain of CSDE1 and the RBD3 domain of SXL [70]. The mutual connection of “CSDE1/SXL/msl-2” played an irreplaceable role in dosage compensation by reprogramming msl-2 translation. Another example is that CSDE1 is involved in the autoregulation of PABP mRNA [51]. CSDE1 can bind both PABP protein and the PABP ARS element directly. However, CSDE1 alone has a lower binding capacity for the ARS than it does when it is acting together with either PABP or IMP1. The presence of PABP can stimulate CSDE1 binding on ARS in a dose-dependent manner. Thus, CSDE1 binds to PABP mRNA and forms a complex with PABP protein during this autoregulation process, and this complex formation results in the repression of PABP expression at the translation level in HeLa cells [51]. Considering the significance of PABP in translation initiation, this mutual connection of “CSDE1/PABP/PABP mRNA” has a crucial role in translation initiation, especially in a cap-dependent manner. The role of CSDE1 in mCRD-mediated mRNA turnover in NIH3T3 and HeLa cells also supports this mechanism [62]. Deadenylation (shortening of the poly(A) tail) of mRNA is the rate-limiting step of mRNA decay and a necessary first step coupled to translation. The mCRD, which is a sequence in the protein-coding region of c-fos mRNA, directs rapid decay of mRNA via deadenylation. CSDE1 binds to the mCRD and forms a complex with four proteins: PABP, PABP-interacting protein 1 (PAIP-1), heterogeneous nuclear ribonucleoprotein R (hnRNP R) and NS1-associated protein 1 (NSAP1) [62]. These proteins prevent deadenylation and RNA decay prior to translation. During the formation of this complex, CSDE1 and PABP bind to the mCRD and the poly(A) tail of c-fos mRNA, respectively. Thus, the CSDE1-PABP interaction built a “bridge” between the c-fos mCRD and its poly(A) tail. This “bridge” is necessary for “CSDE1/protein complex/c-fos” complex formation, which maintains c-fos mRNA stability. This connection reveals a new mechanism for controlling cell growth and differentiation.

Mechanisms similar to this mutual connection have been reported for some other RBPs in previous investigations. The Ccr4-Not complex controls mRNA decay and translation efficiency by removing mRNA poly(A) tails [73]. The complex consists of a number of proteins, mainly Ccr4, Caf1, Caf40, Caf130, Not1, Not2, Not3, Not4, Not5, Not10 and Not11. Shortening of the 3’ poly(A) tail (deadenylation) causes cleavage of the 5’ m7GpppN cap (decapping) and results in translational repression [74]. The Ccr4-Not complex can be recruited by RBPs to the 3’UTR of target mRNAs to promote mRNA decay. For example, the RBP Mpt5p, which is a member of the PUF protein family, controls HO mRNA stability by this mechanism. Mpt5p recruits the Ccr4-Not complex to the HO mRNA 3’UTR to stimulate deadenylation of HO mRNA by binding to Caf1 [75]. The “Mpt5p/Ccr4-Not complex/HO mRNA” consists of mutual connections. Another example is that the RBPs Roquin1 and Roquin2 promote target mRNA degradation by recruiting the Ccr4-Not complex [76]. In addition, the mechanism of some RBPs stimulating translation by recruiting PABP to the RNA poly(A) tail is similar to this mutual connection. As discussed above, PABP binds to a poly(A) tail that has eIFs at the 5’ end. PABP then brings the mRNA ends into close proximity to effectively circularize the mRNA and promote translation. For example, the DAZL family of proteins, which are germ-cell-specific RNA-binding proteins that are essential for gametogenesis, stimulate translation of target mRNAs by recruiting PABPs to the poly(A) tail [77]. Similarly, the “DAZL/PABP/target mRNAs” form a mutual connection. In summary, for the mutual connection model, CSDE1 enhances RNA-protein interactions by interacting with them both. This model is the most important and widely existing mechanism in CSDE1-mediated translation initiation reprogramming.

### Facilitating connections

Some translation regulators can bind to RNA. However, some translational regulators need the help of CSDE1, which acts as a pioneer, paving the way for their binding. With this mechanism, CSDE1 facilitates the binding of regulators and RNAs rather than interacting with regulators directly. CSDE1 changes the structure of bound RNA and makes it more accessible for other protein regulators to bind.

CSDE1 mediates both cellular and virus IRES activity based on this mechanism. Among these different mechanisms, the role of CSDE1 in regulating Apaf-1 and HRV-2 translation initiation is the most widely studied [39, 42]. Both Apaf-1 and HRV-2 mRNA contain IRES elements. Two proteins, CSDE1 and PTB, bind to the IRES elements separately to make the ribosome loading site in the IRES more accessible. Then, CSDE1 and PTB promote IRES-mediated ribosome recruitment and facilitate translation initiation of the bound mRNAs [39, 42]. For the cellular IRES of Apaf-1, CSDE1 first binds to a purine-rich region within a stem-loop structure [39]. This CSDE1 binding forms a structure that permits PTB binding on another exposed loop in the Apaf-1 IRES. Thus, CSDE1 and PTB both bind to the Apaf-1 IRES and promote IRES-dependent translation initiation [39]. For HRV-2, PTB neither interacts with CSDE1 nor affects the binding of CSDE1 to HRV-2 [42]. However, the interaction of PTB with HRV-2 is affected by CSDE1 and HRV-2 binding. In vitro assays showed that CSDE1 bound to subdomains 2 and 5 of the HRV-2 IRES and brought them close together to change the IRES structure [42]. This paved the way for PTB to easily bind. Generally, CSDE1 facilitates PTB binding of Apaf-1 and HRV-2 mRNA, meaning that CSDE1 promotes their translation initiation together with PTB. This facilitating connection mode also exists in noncoding RNAs. The protein Maleless (MLE) and two noncoding RNAs (roX1 and roX2) are components of the dosage compensation complex (DCC) [78]. In dosage compensation, CSDE1 facilitated the binding of MLE to the two roX RNAs and subsequently enhances MLE-roX interaction, which promoted DCC assembly on the chromosome in male Drosophila [79]. This facilitated connection mode is an interesting mechanism. Although CSDE1 cannot bind with these protein regulators, CSDE1 is essential for the regulator binding to the RNAs [42]. Although CSDE1 and the protein regulators bind at two different positions of the RNA with long distances between them, they can still work together to reprogram mRNA translation [39, 42].

**Fig. 2:**
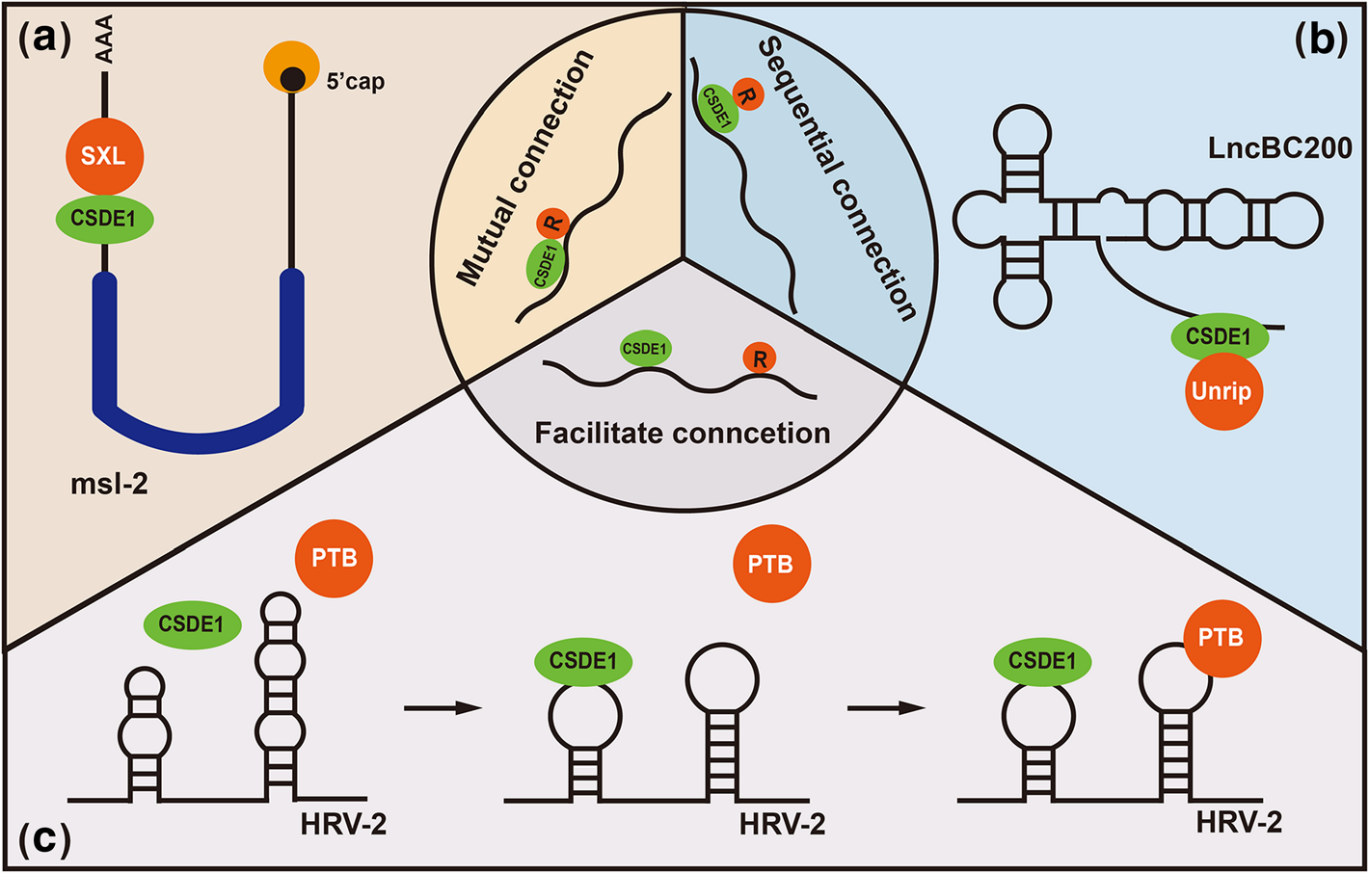
The “protein-RNA connector” model. The “protein-RNA connector” model can be used to explain the bidirectional role of CSDE1 in translational reprogramming. Based on the interactions among CSDE1, regulators and RNAs, this model can be further divided into three forms: sequential connection, mutual connection and facilitating connection. **a** For sequential connection, CSDE1 mediates a protein regulator binding indirectly to RNA. As an example, Unrip binds indirectly to lncBC200 via interacting with CSDE1, which enhances the abundance of lncBC200. **b** For mutual connection, a complex forms among RNA, CSDE1 and protein regulator. As an example, CSDE1, SXL and msl-2 all bind to one another to repress the translation of msl-2. **c** To facilitate connections, CSDE1 binds to RNA to enable the binding of other regulators. As an example, CSDE1 binds to HRV-2 mRNA and changes its structure to facilitate PTB binding, which promotes the translation of HRV-2

In summary, CSDE1 acts as a “protein-RNA connector” in bidirectional reprogramming by three different mechanisms (Fig. 2). As indicated above, the mutual connection mode is the most common. In this mechanism, CSDE1, protein regulators and RNAs bind tightly with each other and form a protein-RNA complex to regulate RNA translation and stability [14, 62]. The facilitating connection mode is the second most common mechanism. In this mechanism, CSDE1 facilitates the binding of other regulators to RNA by adjusting the IRES structure [39, 42]. In a few cases, CSDE1 connects indirect regulators with their target RNAs based on the sequential connection mode [64]. For all modes, CSDE1 binds RNAs directly and cooperates with other regulators in direct or indirect ways. CSDE1 connects different RNAs with their regulators to achieve different purposes (Table 2). The complex of CSDE1, regulator and RNA enable reprogramming of the translation initiation in different directions for each transcript [11, 14]. Thus, CSDE1 plays a bidirectional regulatory role in different biological processes.

**Table 2:** The components and function of CSDE1 in the “Protein-RNA connector” model

Connection models	Protein regulators	RNA	CSDE1 function	Reprogramming directions	Cells	References
Sequential	Unrip	BC200	RNA stability	↑	MCF-7	[64]
Mutual	SXL	Msl-2	Translation	↓	Drosophila female	[14, 69, 70]
	IMP1,PABP	PABP	Translation	↓	HeLa	[51, 54]
	PABP, PAIP-1, hnRNP D, NSAP1	c-fos	RNA stability	↑	NIH3T3; HeLa	[62]
Facilitate	PTB	Apaf-1	Translation	↑	COS7, MRC5, SY5Y, BALB/c	[11]
	PTB	HRV-2	Translation	↑	HeLa	[42]
	PTB	CSDE1	Translation	↓	Mouse ES	[48]

## Role of CSDE1 in human disease

As a critical factor in translational reprogramming, CSDE1 controls the translation of a number of mRNAs that are correlated with various diseases. Previous studies suggest that a pathological status can be reprogrammed by CSDE1 via changing translation of a subset of mRNAs. CSDE1 is emerging as a potential drug target and prognostic biomarker for some diseases (Table 1).

### Cancers

Cancers are the most widely and thoroughly investigated diseases related to CSDE1. CSDE1 has been shown to be a potential drug target, prognostic biomarker and anticancer drug sensitizer for various cancers.

CSDE1 is an oncogene in melanoma, and suppressing CSDE1 can reduce tumour malignancy in both cancer cells and animal models. CSDE1 expression is higher in a variety of melanoma cell lines, including SK-Mel-19, SK-Mel-29, and SK-Mel-103, than in normal melanocytes [15]. In patients, CSDE1 was also higher in both primary and metastatic malignant melanoma tumour samples than it was in benign nevi, as shown by immunohistochemistry (IHC). Additionally, depletion of CSDE1 decreased melanoma tumour growth and metastatic capacity, and overexpression of CSDE1 promoted migration and invasion. Studies in a mouse model further suggested that depletion of CSDE1 strikingly reduced the metastasis to the lung, which is a frequent metastatic site for melanoma [15]. In human colorectal cancer (CRC), CSDE1 promoted cancer cell survival, invasion, and resistance to apoptosis [80]. CSDE1 was also overexpressed by an average of almost 3-fold in tumour samples compared with their adjacent normal tissues in CRC patients. Overexpressed CSDE1 was correlated with poor prognosis in patients. Furthermore, knocking down CSDE1 in CRC cells decreased viability and the migration ratio by approximately 40%. As a treatment, downregulating CSDE1 increased the camptothecin response in CRC-derived cell lines and CRC patients [80].

High expression of CSDE1 was associated with poor prognosis in glioma and pancreatic ductal adenocarcinoma (PDAC) [16]. CSDE1 expression was also significantly higher in glioma cells and tissues than it was in normal human astrocytes and brain tissues. The CSDE1 mRNA expression level was also higher in grade IV glioma than it was in grade II and III patients (*P* < 0.001) according to the data released by the Chinese Glioma Genome Atlas (CGGA). In addition, higher CSDE1 expression was correlated with poorer survival in glioma patients (*P* = 0.0177) [16]. Another recent investigation revealed that low CSDE1 expression was significantly associated with poor outcome in low-grade resected PDAC patients [20]. Based on this study, CSDE1 was used as an independent prognostic biomarker for resectable pancreatic cancer. In addition, for breast cancer, which is the most common cancer affecting women [81], CSDE1 mRNA expression was nearly 10-fold higher in MCF-7 breast cancer cells than in normal cell lines [17].

However, CSDE1 plays an opposing role in some other types of tumours. Pheochromocytomas (PCCs) and paragangliomas (PGLs) are neuroendocrine tumours with low incidence but high mortality [82, 83]. CSDE1 may play a tumour suppressor role in PCCs/PGLs. Loss of function mutations in CSDE1 emerged as a driver of PCC/PGL tumourigenesis. Multi-platform integration, including whole-exome sequencing and mRNA sequencing, revealed that a somatic CSDE1 loss of function mutation drove progression of PCCs/PGLs. CSDE1-mutated tumours exhibited a CSDE1 DNA copy number deletion and inadequate CSDE1 mRNA expression, which supported its role as a tumour suppressor in tumourigenesis [18]. Oral squamous cell carcinoma (OSCC) is one of the most widely occurring cancers and is affected by genetic alternations [84, 85]. A research screen for oxidative stress-related genes revealed that the expression level of CSDE1 was downregulated (median fold change = 0.769) in OSCC (*P* = 0.043) [19].

In addition, CSDE1 was identified as a possible platinum drug sensitizer. Platinum-based chemotherapy is a first-line treatment for epithelial ovarian cancer (EOC). However, approximately 25% of patients are resistant to platinum therapies within 6 months [86]. It was previously reported that some key translation regulation factors played an important role in affecting platinum sensitization [87, 88], and CSDE1 was found to be involved in regulating platinum sensitization of tumours. Silencing CSDE1 caused significant sensitization to platinum in four epithelial ovarian cancer cell lines, SKOV-3, CAOV-3, ES-2 and OVCAR-3. When CSDE1 was silenced, the sensitivity increased by 11 to 50% over what was observed in control cells, which indicated that CSDE1 might be an anticancer drug sensitizer [21]. Furthermore, CSDE1 inhibition increases EOC cell sensitivity to platinum.

### Other diseases

With recent research advances, CSDE1 has been found to play an important role in other diseases, including Diamond-Blackfan anaemia (DBA), autism spectrum disorders (ASDs) and embryonic lethality.

DBA is a rare bone marrow failure disease with a paucity of erythroid precursors [89]. More than half of DBA patients carry mutations in genes encoding ribosomal protein S19 (Rps19) or L11 (Rpl11). A polysome profiling assay showed that the translation of CSDE1 was repressed when Rps19 was lost. In erythroblasts from DBA patients, CSDE1 expression was 3-fold lower than it was in control cells. Decreased CSDE1 expression inhibited erythroid proliferation and differentiation. CSDE1-depleted cells mainly became pyknotic and failed to mature to enucleated erythrocytes [23]. Thus, increasing CSDE1 expression may reverse impaired erythroid proliferation and differentiation.

ASDs are harmful and have a high incidence, with a prevalence of approximately 1–2% [90]. ASD occurrence is largely attributed to genetic variation, especially copy number variations (CNVs) [91]. However, currently known CNVs are mostly rare and account for only a small proportion of cases. Whole exome sequencing of 918 individuals found that a de novo loss of function mutation in CSDE1 was strongly associated with autism [92]. In addition, another genome-wide association study on autism that used two Chinese cohorts for discovery (*n* = 2150) and three data sets of European ancestry populations for validation suggested that CSDE1 was a candidate gene for autism (*P* = 5.51 × 10^− 6^) [22]. These results show that CSDE1 may be one of the causal genes of ASDs, although the detailed mechanism still needs further investigation.

Embryonic development is one of the most important stages of life. In both knockout mice and knockout embryonic stem cells (ECS), CSDE1 was identified as critical for placental development [12, 24]. Lacking CSDE1 resulted in embryonic lethality at mid-gestation. In CSDE1-KO mouse placentas, the spongiotrophoblast and labyrinthine layers showed marked atrophy, and the number of trophoblast giant cells decreased by approximately 60–75% [24]. More importantly, the expression of CSDE1 was significantly higher inhESCs than in their differentiated neural progenitor cells (NPCs) and neuronal counterparts. In addition, CSDE1 protein levels decrease during the hESC differentiation process. CSDE1 expression impairs the neural differentiation of hESCs. Knocking down CSDE1 in hESCs facilitated neural differentiation, and overexpression of CSDE1 impaired neural differentiation by regulating the stability and translation of fatty acid binding protein 7 (FABP7) mRNA as well as the stability of vimentin (VIM) [65]. Therefore, CSDE1 is one of the decisive factors for embryonic development and plays an important role in embryonic reprogramming.

## Conclusions

CSDE1 plays a special bidirectional role in translational reprogramming by regulating the translation initiation and abundance of various RNAs. Among its targets, some RNAs are cell type specific, such as Apaf-1.

Although translational reprogramming is a very complex event, some key factors determine the change of translation during these biological processes [93–95]. We propose that these factors can be considered translational reprogramming points. They determine the expression levels of different mRNAs by changing their translational fate. In our view, CSDE1 acts at special bidirectional translational reprogramming points in various biological processes. The occurrence and development of many diseases are based on the impaired homeostasis of key proteins. As discussed above, CSDE1 determines the expression of many disease-related mRNAs. The bidirectional role of CSDE1 in translation reprogramming contributes to its bidirectional role in cancers: CSDE1 can act as both an oncogene and a tumour suppressor in different tumours. In addition, CSDE1 is emerging as a potential biomarker and therapeutic target in various human diseases. Thus, we propose the “protein-RNA connector” model to explain its bidirectional role. In this molecular model, CSDE1 increases the interaction between RNAs and the regulators, which changes the fate of the RNA, the cell and even the biological processes. This “protein-RNA connector” model helps us to understand the role of CSDE1 in disease reprogramming.

However, there are more areas awaiting investigation. First, the translation reprogramming role of CSDE1 is still not explored at the genome-wide scale. As a connector, CSDE1 should bind to a number of RNAs and proteins to regulate expression at the transcriptome and proteome level. Currently, it is unclear which RNAs are reprogrammed by CSDE1 at the translational level and which protein regulators are connected to specific RNAs by CSDE1 during the translational reprogramming process. Second, the role of CSDE1 in translation homeostasis largely remains unknown. Although CSDE1 bidirectionally reprograms translation within single biological and pathological processes, these processes should maintain homeostasis. Under unbalanced conditions, CSDE1 should be capable of restoring homeostasis by regulating the direction of reprogramming. This role of CSDE1 in stress response needs further investigation. Finally, the role of CSDE1 in human diseases is emerging. It is still unknown how many diseases are correlated with CSDE1-mediated translation reprogramming. The clinical roles for CSDE1 in disease reprogramming, as a predictive biomarker, and as a drug target and sensitizer still need to be confirmed.

## Data Availability

Not applicable.

## Abbreviations

Apaf-1: Apoptotic peptidase activating factor 1
ARS: Autoregulatory sequence
ASD: Autism spectrum disorder
CGGA: Chinese Glioma Genome Atlas
CNVs: Copy number variations
CRC: Colorectal cancer
CSDE1: Cold shock domain containing E1
DBA: Diamond-Blackfan anemia
DCC: Dosage compensation complex
ECS: Embryonic stem cells
eIF4G: Eukaryotic translation initiation factor 4G
EOC: Epithelial ovarian cancer
FABP7: Fatty acid binding protein 7
GATA6: GATA binding protein 6
hESCs: Human embryonic stem cells
Hmbs: Hydroxymethylbilane synthase
hnRNP R: Heterogeneous nuclear ribonucleoprotein R
hnRNPK: Heterogeneous nuclear ribonucleoprotein K
HRV: Human rhinovirus
IHC: Immunohistochemistry
IMP1: Insulin-like growth factor II mRNA binding protein-1
IRES: Internal ribosome entry site
ITAFs: IRES trans-acting factors
lncRNA: Long non-coding RNA
mCRD: Major protein-coding-region determinant
MLE: Maleless
msl-2: Male specific lethal-2
NPC: Neural progenitor cell
NR: Nucleoplasmic reticulum
NSAP1: NS1-associated protein 1
OSCC: Oral squamous cell carcinoma
PABP1: Poly(A)-binding protein 1
PABPC4: Poly(A) binding protein cytoplasmic 4
PAIP-1: PABP-interacting protein 1
PCBP1: Poly (rC) binding protein 1
PCBP2: Poly (rC) binding protein 2
PCCs: Pheochromocytomas
PDAC: Pancreatic ductal adenocarcinoma
PGLs: Paragangliomas
PrE: Primitive endoderm
PTH: Parathyroid hormone
PV: Poliovirus
RBP: RNA-binding protein
RP: Ribosomal protein
RRL: Rabbit reticulocyte lysate
Strap: Serine/threonine kinase receptor associated protein
SXL: Sex lethal
UNR: Upstream of N-RAS
Unrip: UNR-interacting protein
UTR: Untranslated region
VIM: Vimentin
